# Bovine Serum Albumin Protein-Based Liquid Crystal Biosensors for Optical Detection of Toxic Heavy Metals in Water

**DOI:** 10.3390/s20010298

**Published:** 2020-01-05

**Authors:** Noor ul Amin, Humaira Masood Siddiqi, Yang Kun Lin, Zakir Hussain, Nasir Majeed

**Affiliations:** 1 Department of Chemistry, Quaid-i-Azam University, Islamabad 45320, Pakistan; zargey81@yahoo.com (N.u.A.); nasir_majeed86@yahoo.com (N.M.); 2 Department of Chemical & Biomolecular Engineering, National University of Singapore, 4 Engineering Drive 4, Singapore 117585, Singapore; 3 School of Chemical and Materials Engineering (SCME), National University of Sciences and Technology (NUST), H-12, Islamabad 44000, Pakistan; zakir.hussain@scme.nust.edu.pk

**Keywords:** bovine serum albumin, heavy metal ions, liquid crystals, polarized optical microscope

## Abstract

A new methodology involving the use of Bovine Serum Albumin (BSA) as a probe and liquid crystal (LC) as a signal reporter for the detection of heavy metal ions in water at neutral pH was developed. BSA acted as a multi-dentate ligand for the detection of multiple metal ions. The LC sensor was fabricated by immobilizing 3 µg mL^−1^ BSA solution on dimethyloctadecyl-[3-(trimethoxysilyl)propyl]ammonium chloride (DMOAP)-coated glass slides. In the absence of heavy metal ions, a dark optical image was observed, while in their presence, a dark optical image turned to bright. The optical response was characterized by using a polarized optical microscope (POM). The BSA based LC sensor selectively detected toxic metal ions as compared to s block metal ions and ammonium ions in water. Moreover, the limit of detection was found to be very low (i.e., 1 nM) for the developed new biosensor in comparison to reported biosensors.

## 1. Introduction

The term heavy metal ions is employed to any metallic element that has a relatively high density and is toxic or poisonous at low concentrations [1]. Living organisms require specific heavy metal ions such as Cu^2+^ and Zn^2+^ [2] to perform various physiological activities. However, an overdose of these metal ions could lead to some serious health complications and various diseases, such as Parkinson’s and Alzheimer’s [2,3]. Non-essential heavy metal ions such as Pb^2+^ and Hg^2+^ are toxic even at trace levels that can make alterations to the biochemical life cycle and can cause many neurodegenerative diseases in living organisms [4,5]. Aquatic organisms and human beings are exposed to essential and non-essential heavy metal ions through different sources such as water, air, soil, and food [6,7]. Hence, reliable techniques for the detection and quantification of these heavy metal ions on the water surface and within the body fluids of an organism are very important for improving public health and environmental protection [8]. Common methods such as spectroscopy [9], colorimetry [9,10,11], potentiometry [12] and electrochemical techniques [13,14] are employed for these purposes. Despite the fact that these techniques are precise and accurate in identification of toxic metal ions, the major disadvantages associated with them include high cost, longer execution time and need of technically sound workers.

Recently, the use of liquid crystal (LC)-based sensors has been considered as a simple, stable, sensitive and cost-effective detection technique for toxic metal ions [15]. These techniques are based upon the orientational behavior of LC, aligned with the different functional surfaces, that lead to amplified optical signals upon interactions with external stimuli and can be observed with the naked eye [16]. By applying these techniques, a quick and sensitive way of detection of heavy metal ions can be achieved without using complex and expensive instrumentation. Singh, et al. [17] reported Selective detection of Hg^2+^ up to 0.5 µM using thiocarbamate as a chemical probe. Similarly, Chen, et al. [18] also detected Hg^2+^ down to 10 µM using 5-(pyridine-4-yr)-2(5-(pyridine-4-yr)thiophen-yl) diazole. However, these molecular probes were chemically synthesized and used for the detection of a specific metal ion in water and have certain shortcomings such as the chemical synthesis of these probes being complicated; they also cannot be used for the detection and quantifications of heavy metal ions in body fluids. To address these challenges, some research groups used biocompatible and multidentate bio-probes for the identification of targeted metal ions within the body fluids. For instance, Hu, et al. [19] detected Cu^2+^ down to 10 µM via a surface-immobilized urease bio-probe. Similarly, Yang, et al. [15] also used DNA as a bio-probe and LC as a transducer for the identification of Hg^2+^ to the lowest limit of detection of 5 nM.

However, the abovementioned biomolecular probes have been used for the detection of only specific metal ions in solution; sensor arrays which are capable of the detection of multiple metal ions are very rare.

In the present study, we reported a multi-dentate Bovine Serum Albumin (BSA) for the detection of multiple heavy metal ions through LC. To develop the new biosensor, various parameters including surface effect and concentration of BSA probe were optimized and it was tested with different heavy metal ions including Cu^2+^ and Pb^2+^. The efficiency, selectivity, and reproducibility of this sensor was monitored on the basis of the optical results of LC.

## 2. Materials and Methods

Glass slides and Decon-90 were purchased from Fisher scientific (Waltham, MA, USA). BSA, N,N-Dimethyl-N-octadecyl-3-Aminopropyltrimethoxyysilychloride (DMOAP), Ammonium Chloride (NH_4_Cl), Hydrogen Chloride (HCl) were purchased from Sigma Aldrich (Singapore), 4–cyano-4-n-pentylbiphenyl (5 CB) was purchased from Merck (Singapore), while calcium chloride dehydrate (CaCl_2_), sodium Chloride (NaCl), Copper (I) Bromide(CuBr_2_), Copper (II) Sulfate (CuSO_4_), Copper (II) Nitrate [Cu(NO_3_)_2_], Copper (II) Perchlorate [Cu(ClO_4_)_2_.6H_2_O], Mercury (II) Chloride (HgCl_2_), Cobalt (II) Sulphate Co(SO_4_) and Lead (II) Chloride (PbCl_2_) were purchased from Alfa Asar (Singapore).

### 2.1. Preparation of N,N-Dimethyl-N-octadecyl-3-Aminopropyltrimethoxyysilychloride (DMOAP) Modified Surface

Glass slides were cleaned by immersing in a 5% solution of Decon-90 for 12 h. Subsequently, they were rinsed with an excess amount of distilled water and further cleaned in an ultrasonic bath twice, each time for 15 min and dried under a stream of dry nitrogen. Herein, the cleaned glass slides were coated with DMOAP by immersing in an aqueous solution containing 0.1% (v/v) of DMOAP and were agitated for 5 min. Then, the glass slides were rinsed with an excess amount of deionized water to remove the unbound DMOAP and then dried under a stream of nitrogen. The immobilized DMOAP on the surface of the glass slide was cured by placing the glass slides in the vacuum oven at 100 °C for 15 min. Afterward, the glass slides were placed under a UV lamp (254 nm, Model 11S-1, Sigma-Aldrich, St. Louis, MO USA,) for 180 s to activate the DMOAP-coated surface.

### 2.2. Immobilization of Bovine Serum Albumin (BSA) with Heavy Metal Ions

BSA stock solution was prepared by dissolving 1 mg of BSA in 100 mL of an isotonic and non-toxic phosphate buffered saline (PBS) at pH 7 and at 20 °C. Afterward, various concentrations of BSA solutions were prepared and mixed with the metal ion at different concentrations by using a rotor at 6000 rpm. These mixtures were spotted on the activated DMOAP surfaces in an array format by using a spotting robot (AD 1500, Biodot, Irvine, CA, USA). Each spot contained approximately 500 nL of the mixed solution. After an incubation time of 5 h in a humidified chamber, the slides were dried under a stream of nitrogen.

### 2.3. Fabrication of Optical Cell

A hybrid LC cell was fabricated by sandwiching LC between two glass slides by using two strips of spacers (6 μm) and a binder clip. The top glass slide was a DMOAP-coated slide, while the bottom slide contains BSA and heavy metal ions. The LC was drawn into the cavity of the fabricated cell which spread homogeneously by capillary action. The optical appearance of the fabricated LC cell was observed by using a polarizing optical microscope (ECLIPSE LV 100 POL, Nikon, Tokyo, Japan) in the transmission mode. The images were captured using a digital camera mounted on the microscope within an exposure time of 25 ms.

## 3. Results and Discussion

To developed LC and BSA-based biosensors for the detection of heavy metal ions, different parameters comprising interaction of BSA with two different DMOAP surfaces, optimization of BSA concentration, the detection mechanism of the LC/BSA-based sensor, selectivity between heavy metal and s-block metal ions, the limit of detection of different heavy metal ions, reproducibility of optical results and effect of anions on the sensing response of the designed biosensor were evaluated.

### 3.1. The Effect of Modified and Unmodified DMOAP Surfaces on Immobilization of BSA

**Figure 1:**
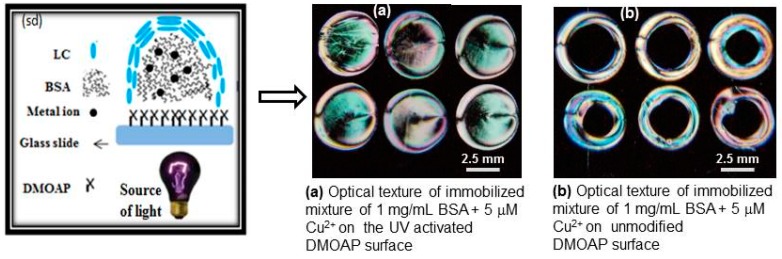
The optical results of an immobilized mixture of 1 mg mL^−1^ Bovine Serum Albumin (BSA) and 5 μΜ Cu^2+^ on (**a**) UV-modified N,N-Dimethyl-N-octadecyl-3-Aminopropyltrimethoxyysilychloride (DMOAP) surface (**b**) unmodified DMOAP surfaces (**sd**) Schematic diagram of the immobilized mixture.

In the previous finding, it was observed that in the immobilization of BSA on solid surfaces, multiple interactive forces have been involved due to the complex structure of BSA, as it contains multi-functional groups [20]. In order to evaluate this observation, a mixture of 1 mg mL^−1^ BSA and 5 µM of Cu^2+^ was immobilized upon unmodified and UV-modified DMOAP-coated glass slide surfaces and characterized through polarized optical microscope (POM) using LC as a transducer. The unmodified DMOAP-coated surface has only an alkyl group at its terminus, whereas in the UV-modified DMOAP surfaces, both aldehydic and carboxylic functional groups were generated under different exposure times [21]. The optical images of LC given in Figure 1a revealed that the UV-activated DMOAP surface adsorbed greater amounts of BSA as compared to the unmodified DMOAP-coated surface shown in Figure 1b. The optical results suggest that the greater surface coverage by BSA might be due to the involvement of strong electrostatic, intermolecular and covalent interactions between the BSA active functional groups and modified DMOAP interfaces [22]. While the lower amount of BSA adsorbed on the unmodified DMOAP surface would be due to the weak non-polar interactions.

Furthermore, the given schematic diagram (Figure 1sd) shows that the mixture of 1 mg mL^−1^ BSA and 5 µM Cu^2+^ ions adsorbed on the aforementioned surfaces is randomly distributed and the coated LC molecules are scattered in a disordered form. When the LC cell was made from them and examined under cross polarizers, colored optical images were obtained.

### 3.2. Optimization of BSA Concentrations

**Figure 2:**
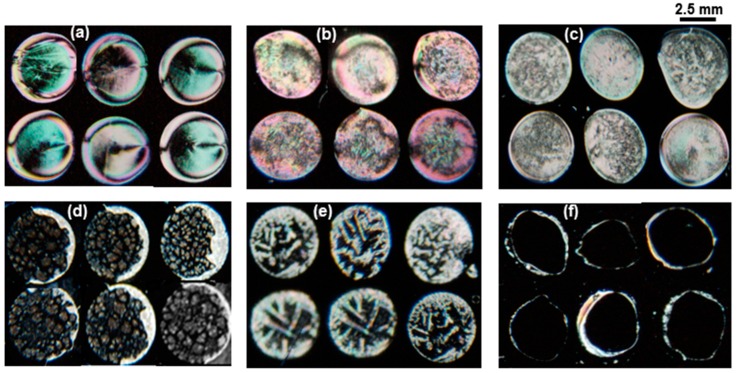
Optical images of liquid crystal (LC) of 5 µΜ (**a**–**d**) and 10 nM (**e**) of Cu^2+^ mixed with various concentrations of BSA immobilized on modified DMOAP surfaces. Whereas the optical image (**f**) has been taken without Cu^2+^.

To get better a signal-to-noise ratio of the biosensor for heavy metal ions detection, optimization of the BSA concentration is required. Thus, various concentrations of BSA protein were mixed with 5 μM of Cu^2+^ ion and immobilized on the UV-modified surface. Then, LC cells were prepared and viewed under a POM and the optical images of LC were obtained as shown in Figure 2. From the POM images, it was observed that the color of LC changed with decreasing BSA concentration. During the optimization of BSA concentration, granular spotty optical textures were obtained at 50 μg mL^−1^ BSA with 5 µM Cu^2+^. However, when the same concentration of BSA was tested for the detection of lower ranges (beyond 5 µM) of metal ions, no detection was observed. When the BSA concentration was further decreased to 3 µg mL^−1^ and tested with various concentrations (1 mM to 1 nM) of heavy metal ions, an improvement in the detection limit of metal ions (up to 1 nM) was observed. From this experiment, the optimized concentration of the BSA probe was found to be 3 µg mL^−1^.

From the color changes observed in the optical images of LC, it was speculated that the BSA would adsorb in a different concentration on the modified DMOAP-coated surface and thus change the surface topology to a different level. Further, when these surfaces were coated with a thin layer of LC, they disrupted the orientation profile of LC to a different extent and, as a result, various colored optical textures of LC were obtained. At very low concentrations, the BSA could get aggregated with the heavy metal ions and patterned the surfaces in such a way at which the LC molecule would be tilted and aligned and, consequently, bright optical images were obtained. This phenomenon can be used as a mechanism to detect Cu^2+^ with the naked eye. The optical images of LC suggested that the BSA molecule at high concentrations could not be aggregated as much as it aggregated at lower concentrations with the 5 µM of Cu^2+^ ions.

One possible explanation for this investigation is that the BSA molecule at higher concentrations could not interact with the metal ions, probably due to the existence of strong intermolecular interactions among and between BSA molecules and, thus, the availability of its binding cavities to form chelating complexes with metal ions is reduced [23]. In contrast, upon immobilization of a mixture of a lower concentration (3 μg mL^−1^) of BSA and Cu^2+^ ion (up to 1 nM), bright granular optical textures of LC appeared suggesting that the BSA molecule could interact and be aggregated upon addition of heavy metal ions. It was already reported that at lower BSA concentrations, various types of intermolecular interactions were disrupted due to extra hydrogen bonding with water that ultimately led to an unfolding state [24]. Finally, it was concluded that the probability of aggregates formed with the more exposed functional ligands of unfolded protein would be much higher than that of hidden active sites of the folded native state.

### 3.3. Detection Mechanism of the Liquid Crystal (LC)/BSA Based Sensor

**Figure 3:**
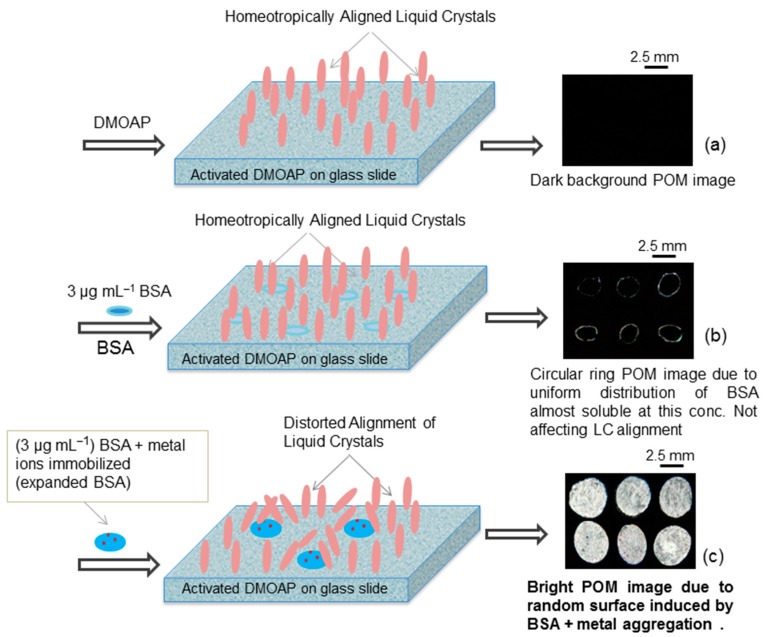
Schematic representation of detection mechanism of the sensing system designed for the detection of metal ions (**a**) DMOAP-coated glass slide polarized optical microscope (POM) dark image (**b**) Immobilized (3 μg mL^−1^) BSA without metal ions on a DMOAP-coated glass slide POM image showing negligible distortion in LC pattern as this amount is soluble in water and is uniformly distributed on DMOAP and (**c**) BSA with metal ions on DMOAP-coated glass slide showing distorted alignment of LC induced by BSA + metal aggregation showing bright POM image.

Figure 3 illustrates the proposed detection mechanism of the LC-based biosensor which is used to detect heavy metal ions in water at neutral pH. The solutions containing BSA were dispensed on glass slides coated with the UV-modified dimethyloctadecyl-[3-(trimethoxysilyl)propyl]ammonium chloride (DMOAP) and nitrogen was blow dried. Then, 10 µL of LC was employed on a glass slide using a micropipette. The BSA without heavy metal ions showed dark optical images due to the homeotropic (perpendicular) alignment of LC, suggesting that the BSA did not change the topology of the modified surface as it is completely soluble at this concentration (Figure 3b). However, The BSA with heavy metal ions gives bright optical images due to the distorted orientation of LC molecules (Figure 3c). The detection mechanism can be supported by a recently reported research article [25].

### 3.4. The Selectivity and Limit of Detection of BSA Based LC Sensor

Interaction of BSA with heavy metals strongly affects both selectivity and limit of detection. Some metal ions can strongly associate, while others show low affinity towards BSA [26]. Upon addition of heavy metal ions at high concentration to BSA, bright granular textures are obtained which in turn reveal their significant interaction with BSA, whereas sodium and ammonium ions are unable to give such textures even at very high concentration. Based on this observation, we claim that the BSA based LC sensor is selective towards heavy metals.

**Figure 4:**
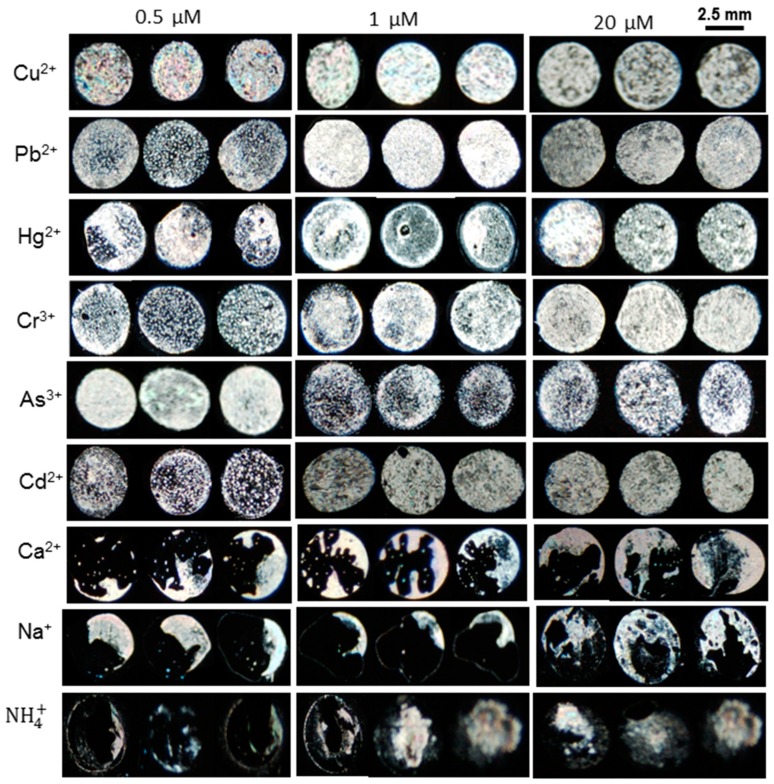
BSA probe shows the selective detection of heavy metals.

The selectivity test was performed by immobilizing various mixtures of 3 µg mL^−1^ BSA with the metal salts at various concentrations (20 µM, 1 µM, and 0.5 µM) on the UV-modified DMOAP surfaces. The LC cell was prepared from each concentration and then examined under the cross polarizers of POM. The non-uniform dark optical textures of LC were observed upon addition of different concentrations of metal ions like Na^+^ and Ca^2+^ and ammonium ions, respectively. The dark optical images revealed that these cations did not show effective interaction with BSA and, consequently, the homeotropic alignment of LC almost remains unchanged. However, fully bright optical images of LC were obtained by incorporating heavy metals such as Cu^2+^, Pb^2+^, Hg^2+^, As^3+^, Co^2+^, and Cr^3+^ into 3 µg mL^−1^ BSA. Thus, the bright optical images indicate that heavy metal cations show effective interaction with BSA (Figure 4). The possible explanation for this finding is that the metal ions and ammonium ions can only be bound to BSA via monodentate mode and would not form strong coordinating or chelating complexes. However, the heavy metal ions form strong chelating complexes with BSA. From the above findings, it can be concluded that the optimized BSA probe at neutral pH is highly selective towards heavy metal ions.

**Figure 5:**
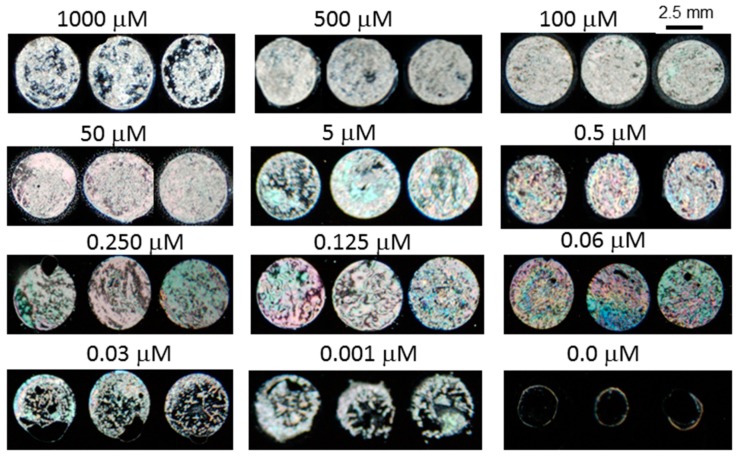
Optical images of LC immobilized mixtures of various Cu^2+^ ion concentration with 3 μg mL^−1^ BSA.

The sensitivity of the designed LC/BSA sensor was investigated with various concentrations of different heavy metal ions. Among the heavy metal ions, Cu^2+^ was first investigated in a way where different concentrations of Cu^2+^ ions (1 mM to 1 nM) with 3 μg mL^−1^ BSA were immobilized on the modified DMOAP surface followed by POM imaging using LC as a transducer. Figure 5 shows the sensitivity of BSA-based detection with Cu^2+^ ions. At a higher concentration of Cu^2+^ ions, the BSA sensor showed bright optical images, while at the lower concentration of copper ions, the brightness of optical images slowly faded away and finally became dark beyond 1 nM. Thus, the acquired optical results indicate that the LC-based optical sensor has a detection limit as low as 1 nM for copper ions.

**Figure 6:**
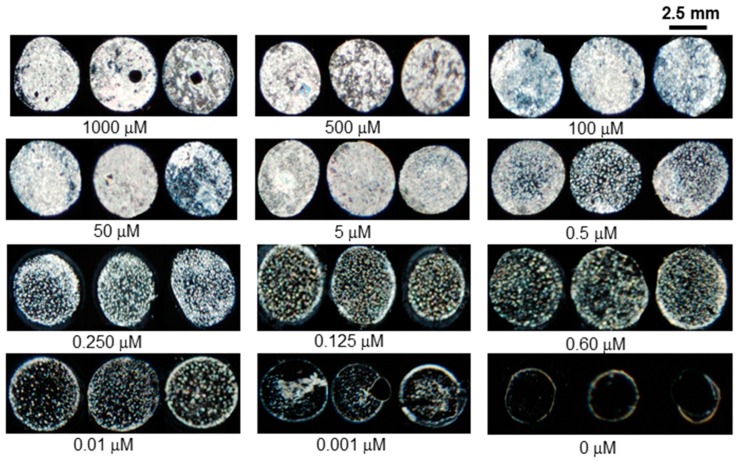
Optical images of LC supported on surfaces with immobilized mixtures of different concentrations of Pb^2+^ ions with 3 μg mL^−1^of BSA.

It has been reported that BSA contains distinct binding sites for different metal ions, such as oxygen and nitrogen atoms present in BSA interact with Pb^2+^ and other heavy metals ions [27]. Similarly, the sensitivity of the designed sensor was investigated with other heavy metal ions including Pb^2+^ by using the same protocol, which was used for the detection of Cu^2+^ ions. In this demonstration (Figure 6), fully bright optical images were appeared by incorporating high concentrations (1 mM, 0.5 mM, 100 µM and 5 µM) of Pb^2+^ ions into 3 μg mL^−1^ BSA. Upon addition of lower concentrations (1 µM to 10 nM) of Pb^2+^ into 3 μg mL^−1^ BSA, bright spotty optical images were observed, whereas the LC optical image of a mixture of 1 nM Pb^2+^ and 3 µg mL^−1^ BSA almost had the same dark appearance as it had in the absence of Pb^2+^.

**Figure 7:**
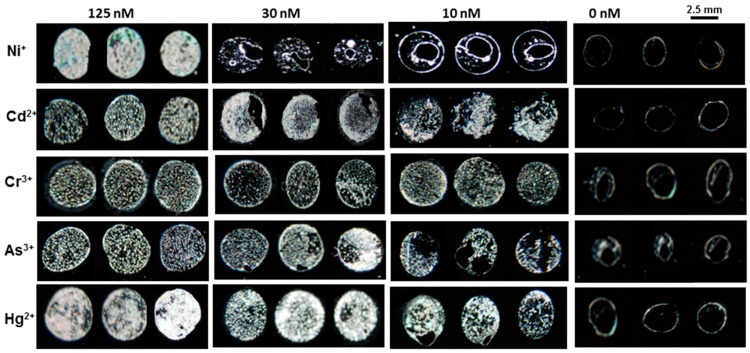
Images show the limit of detection of heavy metals ions with 3 μg mL^−1^ of BSA.

After testing the LC sensor with Cu^2+^ and Pb^2+^ it was clearly revealed that BSA has a high binding affinity with copper as compared to lead and other heavy metal ions. The lower detection limit of copper ions might be due to the fact that BSA had distinct binding sites for Cu^2+^ ions at the N-terminus containing histidyl and aspartyl side residues which have a greater binding affinity with Cu^2+^ ion as compared to other heavy metal ions [28]. The LC/BSA-based sensor was capable of identifying 1 nM of Cu^2+^, whereas the detection limit of other heavy metal ions such as Pb^2+^, Cr^3+^, Hg^2+^,As^3+^and Ni^2+^was in the range of 125 nM to 10 nM at neutral pH as given in Figure 7. The detection limit for different heavy metal ions in the literature was reported in the range of 10 µM to 5 nM using different probes, as summarized in Table 1.

### 3.5. Effect of Anions on Sensing Response of LC Sensor

BSA contains a large number of charged residues (Lys, Cys, His, Glu, and Asp) with potential binding sites, and it has a strong affinity for attracting many anionic moieties [29]. Additionally, in previous studies, it was found that a chaotropic hydrated anion in solution affects the alignment of LC [30].

The BSA molecule at pH 7 becomes negatively charged [31]. The optical signal depends on the interaction of anions with negatively charged BSA at neutral pH. The anions would not bind with BSA and no optical response is observed at low or high concentrations of anions.

**Figure 8:**
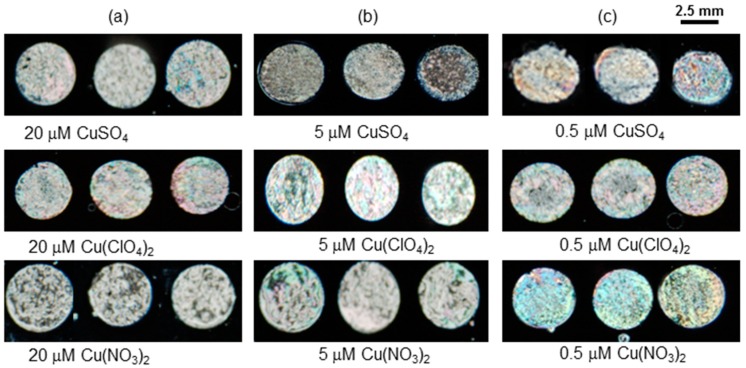
The optical textures of immobilized mixture of 3 μg mL^−1^ BSA and Cu(NO_3_)_2_, Cu(ClO_4_)_2_ and CuSO_4_ at various concentrations.

In order to evaluate the aforementioned observations, different copper salts (CuBr_2_, CuClO_4_, and Cu(SO_4_)_2_) at various concentrations (20 µM, 5 µM, and 0.5 µM) were mixed with 3 µg mL^−1^ BSA and then immobilized on modified DMOAP surfaces. The optical results shown in Figure 8 for each anionic salt (20 µM, 5 µM, and 0.5 µM) revealed that there is no obvious difference in the optical textures of different anionic salts. Therefore, it was concluded that various anions had no influence on the sensing response of the 3 µg mL^−1^ BSA probe.

### 3.6. Reproducability of BSA Based LC Sensor

**Figure 9:**
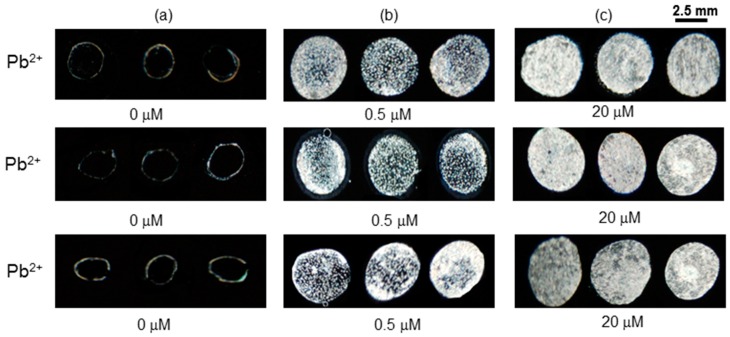
Optical images of LC of immobilized mixtures of Pb^2+^ (**a**) 0 µM (**b**) 0.5 µM and (**c**) 20 µM with 3 μg mL^−1^ BSA.

The LC optical results shown in Figure 9 depict the reproducibility of the sensor in the absence (Figure 9a) and in presence of Pb^2+^ ions at low and high concentrations (Figure 9b,c). In a series of experiments, the optical images remained dark (granular spotty with 0.5 µM and bright with 20 µM) in the absence of Pb^2+^ ions in water at the same sensing conditions. Similar results were also observed for real samples containing heavy metal ions. From this study, it was concluded that the optical images of the BSA-based LC sensor remain unchanged by testing each concentration multiple times and are quite reproducible.

## 4. Conclusions

In this work, we presented a simple and reliable strategy for the real-time detection of heavy metal ions using 3 μg mL^−1^ BSA as a probe and LC as a transducer. We found that an immobilized mixture of 1 mg mL^−1^ BSA and 5 µM Cu^2+^ ions shows significant covalent or non-covalent interactions with the modified DMOAP-coated surface. The concentration of BSA was optimized by immobilization of its various concentrations with 5 µM Cu^2+^ ions. BSA optimization was confirmed from optical imaging and it was found that 3 µg mL^−1^ BSA has a detection limit for heavy metal ions that is as low as 1 nM. The sensing response of the 3 μg mL^−1^ BSA probe was investigated with various normal and toxic heavy metals. The optical images clearly showed that s-block metal ions have no effect on the sensing of the BSA probe; this might be due to the unavailability of unfilled *d*-orbitals, which is a key contributor to the formation of chelating complexes with BSA active sites. However, the core factor in an anionic effect might be the strong hydration of the anions and their subsequent removal along with water under the stream of dry nitrogen. It was also found that the BSA and LC sensor is more sensitive towards copper ions as compared to other heavy metal ions. The BSA-based optical sensor is reproducible and gives a real-time detection of heavy metal ions. The results presented in this article highlighted the feasibility of designing a protein-based sensor for the detection of heavy metal ions using LC as a transducer.

## Figures and Tables

**Table 1:** Shows the detection limits of different heavy metal ions in the literature and in the current study.

Transducer	Probe	Detection Limit	Metal Ions	Ref.
LC	Thiodiacarbamate	0.5 µM	Hg^2+^	[17]
LC	5-(pyridine-4-yl)-2(5-(pyridine-4-yl)thiophen-yl)diazole	10 µM	Hg^2+^	[18]
LC	Urease	10 µM	Cu^2+^	[19]
LC	DNA	5 nM	Hg^2+^	[15]
LC	BSA	1 nM	Cu^2+^	This work
LC	BSA	10 nM	Pb^2+^	This work
